# Potential Impact of Environmental Pollution by Human Antivirals on Avian Influenza Virus Evolution

**DOI:** 10.3390/ani13071127

**Published:** 2023-03-23

**Authors:** Ugo Ala, Paolo Bajardi, Mario Giacobini, Luigi Bertolotti

**Affiliations:** Department of Veterinary Sciences, University of Torino, 10095 Grugliasco, TO, Italy

**Keywords:** oseltamivir, influenza, H5N1, evolution, pollution

## Abstract

**Simple Summary:**

The massive use, often without any real rationale, of antivirals such as Oseltamivir has as a side effect a significant increase in environmental pollution levels. The effect of the spread of these drugs, especially in aquatic basins, has manifested itself in changes in animal behavior with consequent implications for ecological and evolutionary balances. We focused on the effects that can be detected on avian influenza, which today represents one of the most important viral infectious diseases with possible dramatic pandemic repercussions. We evaluated the evolutionary rate of highly pathogenic H5N1 avian influenza strains and a potentially different evolutionary behavior of the H5N1 2.3 viral clade in the presence of environmental pollution by Oseltamivir.

**Abstract:**

Antiviral (AV) drugs are the main line of defense against pandemic influenza. However, different administration policies are applied in countries with different stocks of AV drugs. These policies lead to different occurrences of drug metabolites in the aquatic environment, altering animal behavior with evolutionary consequences on viruses. The aim of this study was to investigate the potential impact of environmental pollution by human antivirals, such as oseltamivir carboxylate (OC), on the evolutionary rate of avian influenza. We used NA, HA, NP, and MP viral segments from two groups of neighboring countries sharing migratory routes of wild birds and characterized by different AV stockpiles. BEAST analyses were performed using the uncorrelated lognormal clock evolutionary model and the Bayesian skyline tree prior model. The ratios between the rate of evolution of the NA gene and the HA, NP, and MP segments were considered. The two groups of countries were compared by analyzing the differences in the ratio distributions. Our analyses highlighted a possible different behavior in the evolution of H5N1 2.3 clade viral strains when OC environmental pollution is present. In conclusion, the widespread consumption of antivirals and their presence in wastewater could influence the selective pressure on viruses.

## 1. Introduction

Highly pathogenic avian influenza (HPAI) H5N1 proved to be an emerging influenza virus, a member of the Orthomyxoviridae, with a dangerous potential to cross the species barrier and cause human infections [1,2]. Its natural hosts are mainly wild birds able to transmit viral infections to domestic animals, producing large outbreaks and promoting the spread of new viral strains in different host species [3]. The influenza pandemic in 1918 and the Asian H5N1 outbreak in 2005 taught a valuable lesson about the need for global action to face the spread of infections. Vaccination is a precious tool for preventing the disease but antiviral drugs (AVs) support and help diseases control, treatment, and resolution. Non-pharmaceutical measures such as human travel reduction [4] or work and school closures [5] are useful in limiting the spread of infections, but with limited effect, except for extreme situations such as during the SARS-CoV2 pandemic [6]. Nowadays, AVs represent the main line of defense against pandemic influenza, especially in limiting patient hospitalization. Neuraminidase (NA) inhibitors-based antivirals are still currently used both for treatment and prophylaxis of seasonal influenza, with Oseltamivir (Tamiflu^®^, Roche, Basel, Switzerland) being the most administered antiviral drug. In this context, a million doses have been stockpiled worldwide, especially after the 2009 H1N1 pandemic [7]. Although there is little evidence of an association between Oseltamivir use and the emergence of oseltamivir-resistant viruses [8,9] and there is no record of such virus variants being transmitted to humans [10], the emergence of resistant strains would represent a major public health concern, highlighting the importance of considering the consumption of antiviral drugs.

Moreover, the presence of drug metabolites in the aquatic environment has been recognized worldwide as one of the emerging issues contributing to environmental pollution [9,11], and recent findings have demonstrated the potential threat of some drugs altering animal behaviors [12,13] and having specific ecological and evolutionary consequences [8,9,14]. The active metabolite oseltamivir carboxylate (OC) is largely excreted by patients via urine and may then persist in the aquatic ecosystem for several weeks since the normal sewage treatment plants are not able to remove or degrade it [15,16]. Indeed, high concentrations of OC have been detected in rivers and water basins during peak influenza seasons, while tens to hundreds of micrograms per liter are expected to be found in case of a mild or severe pandemic scenario [17]. A previous study showed differences in the evolutionary dynamics of avian H5N1 influenza viruses between countries where poultry vaccination was or was not adopted, highlighting how prophylactic practices can modify the viral evolutionary rate [18]. This scenario can create opportunities for the viruses to evolve into new genetic variants and escape from vaccine or drug protection. Moreover, it was proved how exposure to low concentrations of OC can promote the emergence of resistant viruses [8]. Since birds are the major reservoirs of influenza viruses [19] and it has been recognized that avian influenza can be a candidate for the next animal and human pandemic [20,21], accidental exposure to oseltamivir metabolite may also pose a threat to human health.

The aim of the present study was to investigate the impact of environmental pollution by human antivirals on the evolutionary rate of avian influenza viruses.

## 2. Materials and Methods

We restricted our analysis to neighboring countries sharing common migration routes of wild birds [22] and associated with similar ecological features, such as the presence of forests, wetlands, and aquatic reservoirs. We grouped the avian viral isolates into two cohorts according to antiviral availability in the respective countries, focusing on highly pathogenic H5N1 avian influenza strains. We selected sequences from GenBank deriving from Hong Kong, Japan, and South Korea (large use of OC, “Large Antiviral Stockpile” or “Large AS”), on the one hand, and China, Russia, and Mongolia (limited use of OC, “Limited AS”), on the other, between 2003 and 2011 (Supplementary Material Document S1).

For evaluating the role of OC on AI evolution we considered different genes, giving their role and interaction with the drug. The NA gene segment was included as the main target of OC. HA, NP, and MP were considered because they are not targets of OC and they were used as reference genes for each viral strain to allow a comparison between the two cohorts. We downloaded the NA, HA, NP, and MP segments and we used the WHO reference sequences to identify the clade membership of every isolate [23]. We constructed the uncorrected p similarity matrix for each segment using PAUP software [24]. Considering the HA gene distance matrix, we assigned the clade membership to each isolate identifying the closest reference HA sequence. To exclude reassortment events, isolates were further compared: genetic distance matrices were analyzed to detect nearest neighbor samples, and samples belonging to the same clade were identified. Samples that did not respect clade assignment in one or more segments were excluded because reassortment was suspected. This approach led us to focus only on clade 2.3 (Table 1, Supplementary Material Document S2). The sequences of the four segments for each viral strain were concatenated and aligned respecting the coding frame.

In order to verify if the datasets were comparable, p-distance and ω (dN/dS) were calculated for both the concatenated sequences and the NA gene only using PAUP and the Datamonkey server [25]. BEAST runs were performed using the uncorrelated lognormal clock evolutionary model and Bayesian skyline tree prior model [26]. The BEAST input files included all the information related to the partitioned alignment, and they are available as Supplementary Files (Supplementary Material Document S3). A minimum of 10^8^ generation steps were used to reach chain convergence and sampled every 1000 generations. The results were considered significant if supported by ESS values greater than 100. For every recorded topology, after the burn-in, we collected the evolutionary rate of the four segments and compared the evolutionary rate of NA to the rate of the reference genes, and used it to compare sequences belonging to the different cohorts.

Median evolutionary rates are reported with their 95% highest posterior density (HPD) intervals. Continuous variables, such as the evolutionary rates ratios, are described by median values, as well as first and third quartiles. The one-sample Wilcoxon signed rank test was used to assess the statistical differences between the evolutionary rates ratio medians in the null hypothesis of a median equal to one.

In order to compare the Large and Limited AS countries, 1000 random selections of 100 ratio values were performed for each gene pair (NA/HA, NA/NP, and NA/MP) in both the country cohorts. For example, in the case of NA/HA comparison, we defined as R^HA^_Large_ the set of 100 randomly selected ratios between NA and HA evolutionary rates from the Large AS cohort, and as R^HA^_Limited_ the set of 100 randomly selected ratios between NA and HA evolutionary rates from Limited AS cohort. The two ratio distributions (R^HA^_Large_ and R^HA^_Limited_) were compared using the Wilcoxon rank sum test. This comparison was repeated for each of the 1000 random selections, recording the medians of the two distributions and the significance level (adjusted *p*-values according to Benjamini and Hochberg) of each comparison for each repetition. Finally, the median of the median distributions together with the proportion of adjusted p-values lower than 0.01 are reported in Table 1. The same approach was used for NA/NP and NA/MP comparisons.

## 3. Results

A total of 188 and 124 sequences were selected for the Large and the Limited AS countries respectively (as detailed in Table 1). Genetic features were evaluated showing a very similar behavior (mean concatenated sequences p-distance ± standard deviation: Large AS = 0.023 ± 0.011; Limited AS = 0.025 ± 0.017; mean NA gene sequences: Large AS = 0.032 ± 0.025; Limited AS = 0.029 ± 0.016). In the same way, ω values were very similar, suggesting a similar evolution between cohorts (see Table 1).

The evolutionary rates of each viral segment were estimated through BEAST Bayesian analyses. The resulting log files were visualized using Tracer [27] and analyzed with R statistical software [28]. To assess the chains’ convergence and to select only equally probable topologies, the burn-in values were manually set at 10% and 50% for the Large and Limited AS cohorts, respectively. The obtained evolutionary rates are reported in Table 1.

**Figure 1:**
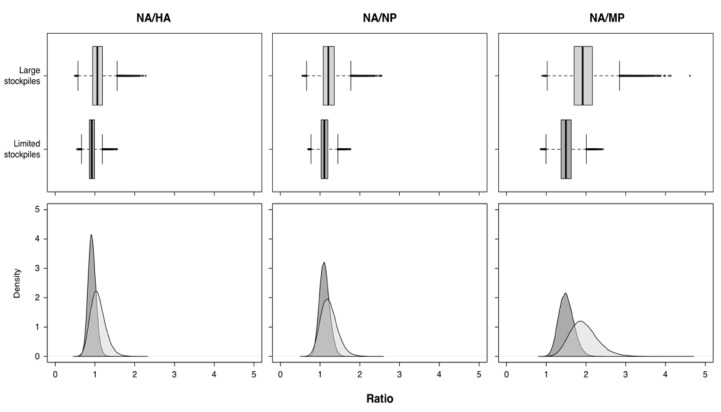
Distribution of the evolutionary rates ratios between NA and the reference genes (HA, NP, and MP from **left** to **right**). Data from countries with large antiviral stockpiles are represented in light gray, whereas those from countries with limited antiviral stockpiles are represented in dark gray.

Our results showed similar evolutionary behavior in the two cohorts (i.e., similar ω values) but larger evolutionary rates of all the viral segments from the Limited AS cohort. The ratios between the NA and the reference genes’ evolutionary rates were calculated for both cohorts. The medians of the ratio distributions were significantly different from 1 in all cases: specifically, all medians were greater than 1 except for NA/HA in the Limited AS cohort, which was significantly lower than 1 (Table 1 and Figure 1).

**Figure 2:**
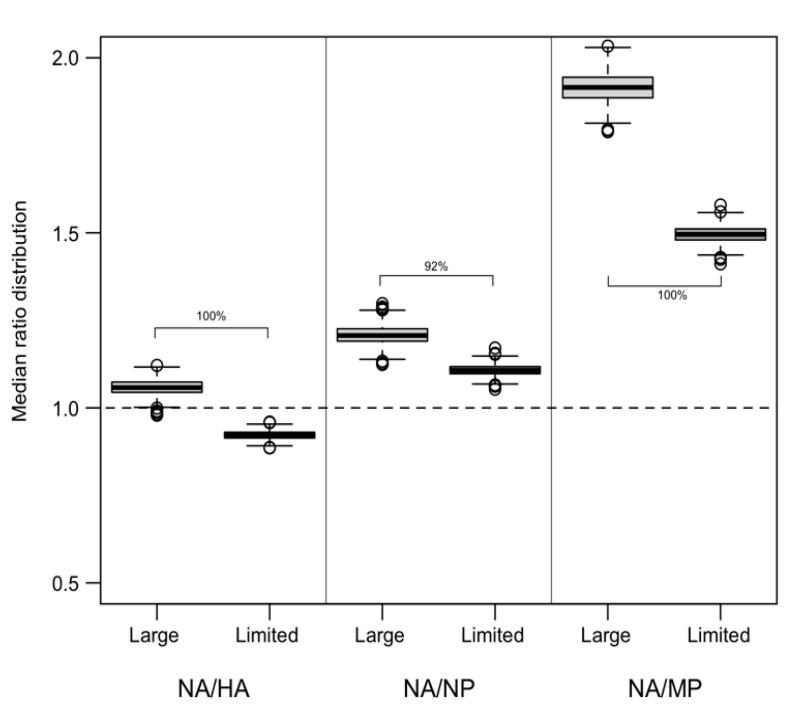
Median ratios distribution obtained from 1000 random extraction procedures (see Materials and Methods section for details). The percentage reported on cohort comparison indicates the proportion of significant (*p* < 0.01) results.

Finally, a total of 100 random ratio values from both cohorts were extracted for each gene pair. The two sample distributions were compared to support the hypothesis of faster evolution of NA in Large AS countries. A thousand repetitions were conducted to statistically support the results. As reported in Figure 2, the results suggest a faster NA evolution of viral strains collected in Large AS countries, showing a global shift of their distributions to higher values. Clear differences are evident if NA/HA and NA/MP comparisons are considered: 100% of the 1000 adjusted p-values were lower than 0.01. A partial overlap of the distributions of median ratios is detectable in the NA/NP case, highlighted by a percentage lower than 100% (92%) of significant results (Figure 2).

## 4. Discussion

The appearance of resistant microorganisms is widely considered the main near-future global health and development threat. Even if attention is strongly focused on bacteria, drug resistance also emerges in viral infections, increasing the risk of uncontrolled epidemic events. It is well known that there are several driving forces in the avian influenza virus, but we cannot exclude a possible role of AV drugs in modulating viral evolution.

Oseltamivir resistance was detected in persons undergoing treatment for influenza A and B infections [29], with the highest frequency of resistance seen in young children after initiation of oseltamivir treatment. Several amino acid changes in the neuraminidase (NA) were responsible for variable degrees of resistance. In the 2007–2008 season, the emergence of widespread oseltamivir-resistant A(H1N1) viruses carrying the H274Y mutation was detected in several countries, with the highest frequency of resistance (up to 67%) seen in Europe [30]. Our analyses suggest a possible similar behavior in the evolution of H5N1 clade 2.3 viral strains if OC environmental pollution is suspected.

In more detail, the evolutionary rate of the NA gene, the target of the OC, looks to be different compared to the other viral gene segments, allowing the possible emergence of resistance to the drug.

Two main aspects are noteworthy. First, the p-distance and ω values of the Limited AS and Large AS cohorts are very similar, strongly suggesting a similar evolutionary behavior between the considered geographical areas, for both NA genes and concatenated alignments. On the other hand, the evolutionary rates calculated in the Limited AS cohort, in line with the previously published data [18], are higher than the ones calculated in the Large AS cohort. This result suggests that a wider viral circulation as well as the absence of a complete control system could push all the viral genes to evolve faster [31].

As a second point, the ratios (NA/HA, NA/NP, and HA/MP) in the Limited AS cohort are interestingly lower than the ratios calculated in the Large AS cohort. This result could be explained by the possible role of OC pollution. Indeed, data from countries with high OC consumption showed a higher median evolutionary rate of NA, the target of OC, compared to other viral genes.

Even if HA showed the highest values in terms of evolutionary rates if cohorts are compared, NA always showed a faster evolutionary rate compared to the reference genes belonging to the same viral genome (ratio greater than 1 except in the HA sequences comparison in the Limited AS cohort; see Table 1). More importantly, the results suggested how the presence of an environmental contaminant could interfere with the evolution of H5N1 influenza viral strains, in particular with its target.

Depending on the choices of the reference gene we either observe a mild–neutral (as in the case of NA/NP) or strong effect, suggesting that the NA segment evolves faster in countries with large antiviral stockpiles.

Even if in the included sequences no H274Y substitution in its neuraminidase protein was present, the high evolutionary rate could push the virus to create new divergent and resistant progeny, as supported both in the experimental condition and by recent studies proving the emergence of resistant viral strains [30].

## 5. Conclusions

Oseltamivir was used worldwide as an antiviral response against influenza outbreaks, even if its efficacy was widely debated [1]. Focusing on the biological aspects, it is clear that all drugs, vaccines, or control measures can change the environmental framework around microorganisms. Like the antimicrobial resistance phenomenon, it is evident how microorganisms can evolve and adapt to new conditions. In this light, it is extremely important to face this problem. Knowledge of pathogen evolution is fundamental for understanding if/how a pathogen is changing and if diagnostic and prophylactic tools are still effective. Our results suggest how new strategies against pathogens should be implemented and improved. A major effort should be made to reduce the use of drugs, increase the biosecurity levels of different communities of both human and animal populations, and improve care for the environment.

## Figures and Tables

**Table 1:** Summary of the results. Description of the two cohorts including values related to selective pressure (SLAC and MEME evaluated with the Datamonkey server); the estimated evolutionary rate of the four segments in the two cohorts (95% HPD range is reported in brackets); ratios between NA and the other segments in the two cohorts (25th and 75th quantiles are reported in brackets); distributions of the medians obtained from 1000 random extractions of ratios of both cohorts, for each gene pair (25th and 75th quantiles are reported in brackets).

		Large AS	Limited AS
Cohort description		Japan (*n* = 39) Hong Kong (*n* = 49) South Korea (*n* = 36) SLAC w: 0.18 MEME w: 0.17 SLAC_NA_ w: 0.20 MEME_NA_ w: 0.18	China (*n* = 177) Russia (*n* = 6) Mongolia (*n* = 5) SLAC w: 0.19 MEME w: 0.18 SLAC_NA_ w: 0.27 MEME_NA_ w: 0.24
Median evolutionary rate sub/site/year x10^−3^ (95% HPD)	NA	1.95 (1.37–2.68)	4.29 (3.57–5.10)
	HA	1.85 (1.29–2.53)	4.65 (3.91–5.49)
	NP	1.62 (1.13–2.23)	3.87 (3.13–4.67)
	MP	1.02 (0.71–1.40)	2.86 (2.26–3.57)
Ratios (25th–75th quantile)	NA/HA	1.059 (0.943–1.190)	0.922 (0.859–0.990)
	NA/NP	1.208 (1.076–1.355)	1.107 (1.026–1.195)
	NA/MP	1.916 (1.702–2.159)	1.495 (1.373–1.627)
Random ratio distribution Median (25th–75th quantile)	NA/HA	1.060 (1.044–1.077)	0.922 (0.914–0.931)
	NA/NP	1.207 (1.190–1.226)	1.107 (1.096–1.117)
	NA/MP	1.915 (1.888–1.945)	1.496 (1.477–1.511)

## Data Availability

Not applicable.

## Supplementary Materials

The following supporting information can be downloaded at: https://www.mdpi.com/article/10.3390/ani13071127/s1, Document S1: Cohorts description [7,32,33,34,35,36,37,38]; Document S2: Sequence accession numbers; Document S3: BEAST input files.
