# Supplementary material for: Potential Impact of Environmental Pollution by Human Antivirals on Avian Influenza Virus Evolution
Source: Animals (Basel). 2023 Mar 23;13(7):1127. doi: 10.3390/ani13071127 (PMC10093092; doi:10.3390/ani13071127)
Supplement: Supplementary file 1 [file animals-13-01127-s001.zip › Document S1.pdf]

SUPPLEMENTARY MATERIAL 1.

The six Countries that were considered (China, Russia, Mongolia, South Korea, Japan, Hong Kong) share the migration route of wild birds and they were divided according to the percentage of the population that could have access to antivirals stockpiles.
Specifically, the first cohort, Limited AS (China, Russia, Mongolia) is characterized by a relative amount of antiviral stockpile lesser than 3%. Instead the Large AS cohort shows a much greater relative amount of antiviral stockpile (larger than 10%).
Lacking detailed scientific publication on this specific topic, we explored other sources of information too.
In particular, regarding

- China
  - according to Table 1 in [A], China has an amount of stockpile corresponding to an *1%* of its population.
- Russia
  - according to a WHO document [B], Avian Influenza H5N1 is fanning out across Russia and Kazakhstan and "Roche has agreed to reserve three million treatment courses (30 million capsules) for up to five years". Considering a population of around 143,000,000 (in 2009) [C], the amount of stockpile would correspond to about *2%* of its population.
- Mongolia
  - as reported by Reuters Agency [D], "The WHO sent over 45,000 doses of Tamiflu to Mongolia" (in 2009) in addition to the 11,000 already there. Considering a population of around 2,700,000 (in 2009) [E], the amount of stockpile would correspond to about *2%* of its population.
- South Korea
  - according to "Action taken across the region to counter swine flu" by The New Humanitarian publication (2009) [F]: "Tamiflu stockpile for 2.5 million people, and is to increase that to *10 percent* of 50 million population"
- Japan
  - according to "Action taken across the region to counter swine flu" by The New Humanitarian publication (2009) [F]: "Tamiflu stockpiles for about 22.5 million, and Relenza stockpile for about 2.68 million people, together covering nearly *20 percent* of population. Local governments have own stockpiles but figures not available"
- Hong Kong
  - according to written reply by the Secretary for Food and Health, Dr York Chow, in the Legislative Council today (January 14, 2009) [G]: "Among the existing stockpile of drugs against avian influenza, 1,003,910 doses have expired, while the remaining 19,646,250 doses are still unexpired". Considering a population of around 7,000,000 (in 2009) [H], the amount of stockpile would correspond to about *280%* of its population.

[A] Ghosh S, Heffernan J. Influenza pandemic waves under various mitigation strategies with 2009 H1N1 as a case study. PLoS One. 2010 Dec 20;5(12):e14307. doi: 10.1371/journal.pone.0014307. PMID: 21187938; PMCID: PMC3004963.
[B] https://apps.who.int/mediacentre/news/releases/2005/pr36/en/index.html
[C] https://en.wikipedia.org/wiki/Demographics_of_Russia
[D] https://www.reuters.com/article/flu-asia/update-1-tamiflu-shipped-to-mongolia-to-fight-h1n1-outbreak-idUKSP7332220091105
[E] https://en.wikipedia.org/wiki/Demographics_of_Mongolia
[F] https://www.thenewhumanitarian.org/report/84164/asia-action-taken-across-region-counter-swine-flu
[G] https://www.info.gov.hk/gia/general/200901/14/P200901140138.htm

[H] https://en.wikipedia.org/wiki/Demographics_of_Hong_Kong
